# Ectopic foci do not co‐locate with ventricular epicardial stretch during early acute regional ischemia in isolated pig hearts

**DOI:** 10.14814/phy2.15492

**Published:** 2022-10-18

**Authors:** Hanyu Zhang, Han Yu, Gregory P. Walcott, Jack M. Rogers

**Affiliations:** ^1^ Department of Biomedical Engineering University of Alabama at Birmingham Birmingham Alabama USA; ^2^ Department of Medicine University of Alabama at Birmingham Birmingham Alabama USA

## Abstract

Ectopic activation during early acute regional ischemia may initiate fatal reentrant arrhythmias. However, the origin of this ectopy remains poorly understood. Studies suggest that systolic stretch arising from dyskinesia in ischemic tissue may cause ectopic depolarization due to cardiac mechanosensitivity. The aim of this study was to investigate the link between mechanical stretch and ectopic electrical activation during early acute regional ischemia. We used a recently developed optical mapping technique capable of simultaneous imaging of mechanical deformation and electrical activation in isolated hearts. Eight domestic swine hearts were prepared in left ventricular working mode (LVW), in which the left ventricle was loaded and contracting. In an additional eight non‐working (NW) hearts, contraction was pharmacologically suppressed with blebbistatin and the left ventricle was not loaded. In both groups, the left anterior descending coronary artery was tied below the first diagonal branch. Positive mechanical stretch (bulging) during systole was observed in the ischemic zones of LVW, but not NW, hearts. During ischemia phase 1a (0–15 min post‐occlusion), LVW hearts had more ectopic beats than NW hearts (median: 19, interquartile range: 10–28 vs. median: 2, interquartile range: 1–6; *p* = 0.02); but the difference during phase 1b (15–60 min post‐occlusion) was not significant (median: 27, interquartile range: 22–42 vs. median: 16, interquartile range: 12–31; *p* = 0.37). Ectopic beats arose preferentially from the ischemic border zone in both groups (*p* < 0.01). In LVW hearts, local mechanical stretch was only occasionally co‐located with ectopic foci (9 of 69 ectopic beats). Despite the higher rate of ectopy observed in LVW hearts during ischemia phase 1a, the ectopic beats generally did not arise by the hypothesized mechanism in which ectopic foci are generated by co‐local epicardial mechanical stretch.

## Graphical abstract

Electrical propagation and epicardial deformation were imaged during acute regional ischemia in isolated pig hearts using a novel optical mapping system. Hearts in left‐ventricular working mode exhibited a higher rate of ectopy than control hearts with suppressed mechanical stretch. However, electromechanical mapping data showed that ectopic foci were only occasionally co‐located with local mechanical stretch, suggesting that mechanically‐induced depolarization of stretched border zone tissue was not the major source of ectopy.

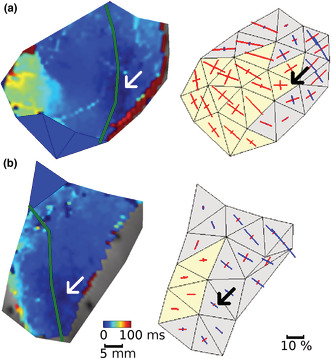

## 1 INTRODUCTION

The formation of potentially fatal reentrant arrhythmias requires two concurrent factors: trigger and substrate (Tse, 2016). During the early stages of coronary occlusion, ectopic activations may act as triggers in the electrophysiologically heterogeneous substrate (Janse et al., 1979) to produce wavebreak leading to arrhythmias such as ventricular fibrillation (VF; Zaitsev et al., 2003) Previous studies indicate that in the first hour following coronary occlusion, there are two phases of frequent ectopic activations, referred to as phase 1a and 1b (Kaplinsky et al., 1979). However, the origin of this ectopy remains poorly understood.

Recent studies have suggested that cardiac mechanosensitivity may play a role in the initiation of post‐occlusion ectopic activations. Mechanosensitivity is an intrinsic property of the heart that exists in isolated heart, isolated cardiac tissue, and isolated cardiac cells (Peyronnet et al., 2016). Mechanical stimulation can modulate cardiac electrical activity and, under certain conditions, can be arrhythmogenic (Franz et al., 1992; Maron & Estes, 2010). Potential mechanisms include the opening of stretch‐activated ion channels (SACs; Craelius et al., 1988; Guharay & Sachs, 1984; Peyronnet et al., 2016), calcium release (Cameron et al., 2020), altered voltage‐gated channel behavior (Morris, 2011) etc. Clinical studies and experimental models have suggested a potential link between mechanical stretch and ectopic activation in ventricular tissue during the early phases of acute regional ischemia (Coronel et al., 2002; Jie et al., 2010; Parker et al., 2004; Siogas et al., 1998). It is possible in principle to pharmacologically block SACs, which could partially clarify the contribution of mechanical stretch to ventricular ectopy. However, such agents may have minimal effect in intact hearts (Cooper & Kohl, 2005; Zhang et al., 2018) or may not be practical in large animal models (Bode et al., 2001; Cooper & Kohl, 2005).

We have developed a novel cardiac optical mapping system that can simultaneously record electrical activity and mechanical deformation in isolated, perfused, large animal hearts (Zhang et al., 2016). In the present study, we use this optical mapping system to investigate the role of mechanical stretch in initiating ectopic activations during the early stages of acute regional ischemia in isolated swine hearts. Specifically, we test the hypotheses that: *I*, mechanically loaded hearts (i.e., in left ventricular working mode) have higher post‐occlusion ectopic activation frequency than unloaded hearts with suppressed contraction; and *II*, post‐occlusion ectopic activations in mechanically loaded hearts arise preferentially from ventricular sites undergoing mechanical stretch.

## 2 METHODS

All animal protocols were approved by the University of Alabama at Birmingham Institutional Animal Care and Use Committee (APN 09818) and were in accordance with the Guide for the Care and Use of Laboratory Animals.

### 2.1 Heart preparation

Isolated hearts from 22 pigs of either sex, weighting 25–35 kg, were studied. Sixteen were assigned to two groups of eight: non‐working (NW) hearts were Langendorff perfused with crystalloid solution. Contraction was suppressed with blebbistatin and the left ventricle was empty. In the left ventricular working hearts (LVW), contraction was not suppressed and the left ventricle pumped perfusate against a hydrostatic pressure load. Three additional Langendorff‐perfused hearts, which were prepared similarly to the NW hearts but without coronary occlusion, were used as a sham group. Three additional working hearts were used to evaluate deformation homogeneity. Heart isolation, perfusion, blebbistatin incubation, and voltage‐sensitive dye loading are detailed in the Supplementary Methods. Anesthesia for the isolation procedure was induced with intramuscular telazol (4.4 mg/kg), xylazine (2.2 mg/kg), and atropine (0.04 mg/kg) and maintained with isoflurane by inhalation in 100% oxygen (1.5%–2.5%). Euthanasia was by exsanguination secondary to heart excision.

### 2.2 Instrumentation

In NW and LVW hearts, anterior left ventricular (LV) epicardial electrical activity and mechanical deformation were measured with our recently developed electromechanical optical mapping system (Zhang et al., 2016). This system uses a combination of motion tracking and excitation ratiometry to obtain membrane potential (*V*_m_) signals in beating hearts. Twenty to thirty black circular markers (2 mm diameter, ~8 mm spacing) were glued to the anterior ventricular epicardium and the hearts were stained with di‐4‐ANEPPS (di4; Biotium) for imaging *V*_m_ propagation. Marker motion was tracked to characterize epicardial deformation and to partially correct motion artifact in *V*_m_ recordings. Excitation ratiometry was used for further motion artifact correction. See the Supplementary Methods for additional information on the optical mapping system.

**FIGURE 1:**
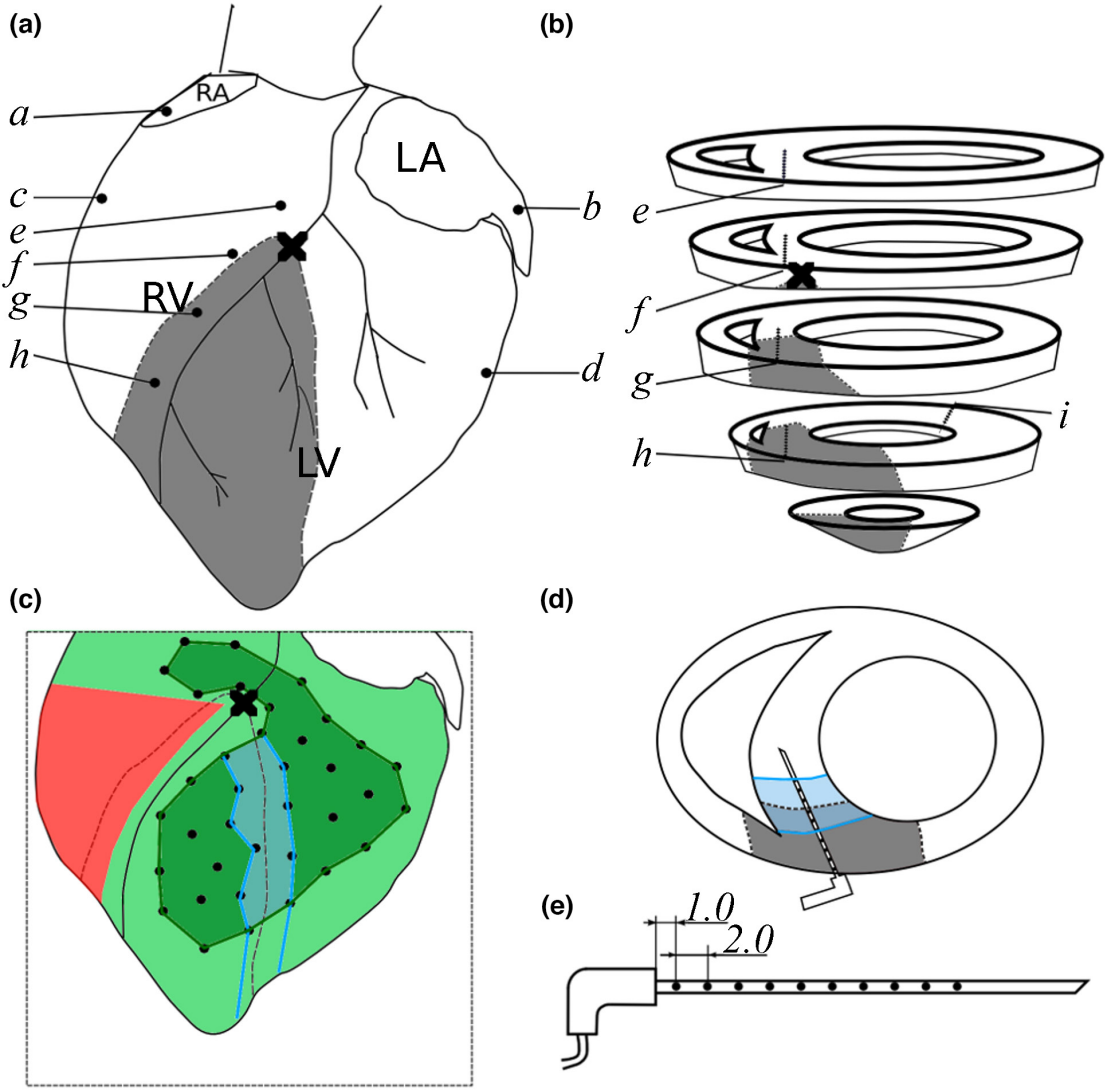
Electrode placement and optical mapping regions. (a) Location of electrodes relative to the anterior epicardium. (b) Location of plunge needles in the septum and posterior LV. (*a*–*d*), subepicardial hook electrodes; (*e*–*i*), plunge needles; black cross, LAD ligation site; gray shading, ischemic zone. (c) Optical mapping regions on the anterior epicardium. Dark green shading, marked region; cyan shading, ischemic border zone (IBZ); light green shading, expanded mapping region. The red region is obscured by electrode cables and is not available for optical mapping. (d) Relative locations of the septal IBZ and a plunge needle electrode. (e) dimensions (in mm) of plunge needle electrode. LAD, left anterior descending coronary artery; LV, left ventricular

In NW and LVW hearts, we also mapped unipolar extracellular potentials from sites that were not imaged by the optical mapping system. Four 10‐channel custom‐made plunge needle electrodes (Rogers et al., 2002) were inserted into the ventricular septum from the anterior side and one plunge needle electrode was inserted into the posterior LV wall. Four electrodes were also hooked to the subepicardium of the right atrium (RA), left atrium, right ventricle (RV) and LV. The unipolar electrogram signals were recorded with an electrical mapping system (Wolf et al., 1993) at 2000 Hz sampling frequency. The electrical mapping system was synchronized with the optical mapping system. The instrumentation setup is illustrated in Figure 1.

### 2.3 Experimental protocols

In LVW, NW, and sham hearts, during the 10‐min interval following di4 loading, the dissolved oxygen level of the coronary effluent was monitored. The perfusion flow rate for NW and sham hearts was adjusted so that the coronary effluent had a similar dissolved oxygen level as that in LVW hearts.

In the NW and LVW hearts, 10‐s‐long baseline data records were acquired with the synchronized optical and electrical mapping systems. Acute regional ischemia was then induced by tying a suture on the left anterior descending coronary artery between the first and second diagonals. Post‐occlusion recording started immediately and continued for 60 min. Hearts were not paced; only spontaneous electrical activity was recorded. In two NW hearts, there were single instances of VF that were successfully defibrillated without interrupting recording. In the sham hearts, we recorded unipolar electrograms from the four subepicardial electrodes for 60 min, but no other electrical or optical mapping was performed.

After acquiring electrical and optical mapping data, the plunge needle electrodes were replaced with Teflon tubes to mark the location of each needle (Dosdall et al., 2007). The hearts were then removed from the mapping apparatus and perfused with fast green dye. The anterior side was photographed to document the epicardial location of the perfusion boundary. Hearts were then sliced transversely at the level of each needle track and photographed to document the location of the septal perfusion boundary and to estimate the locations of plunge needle electrodes relative to the perfusion boundary.

### 2.4 Data processing

#### 2.4.1 Identification of ectopic events

**FIGURE 2:**
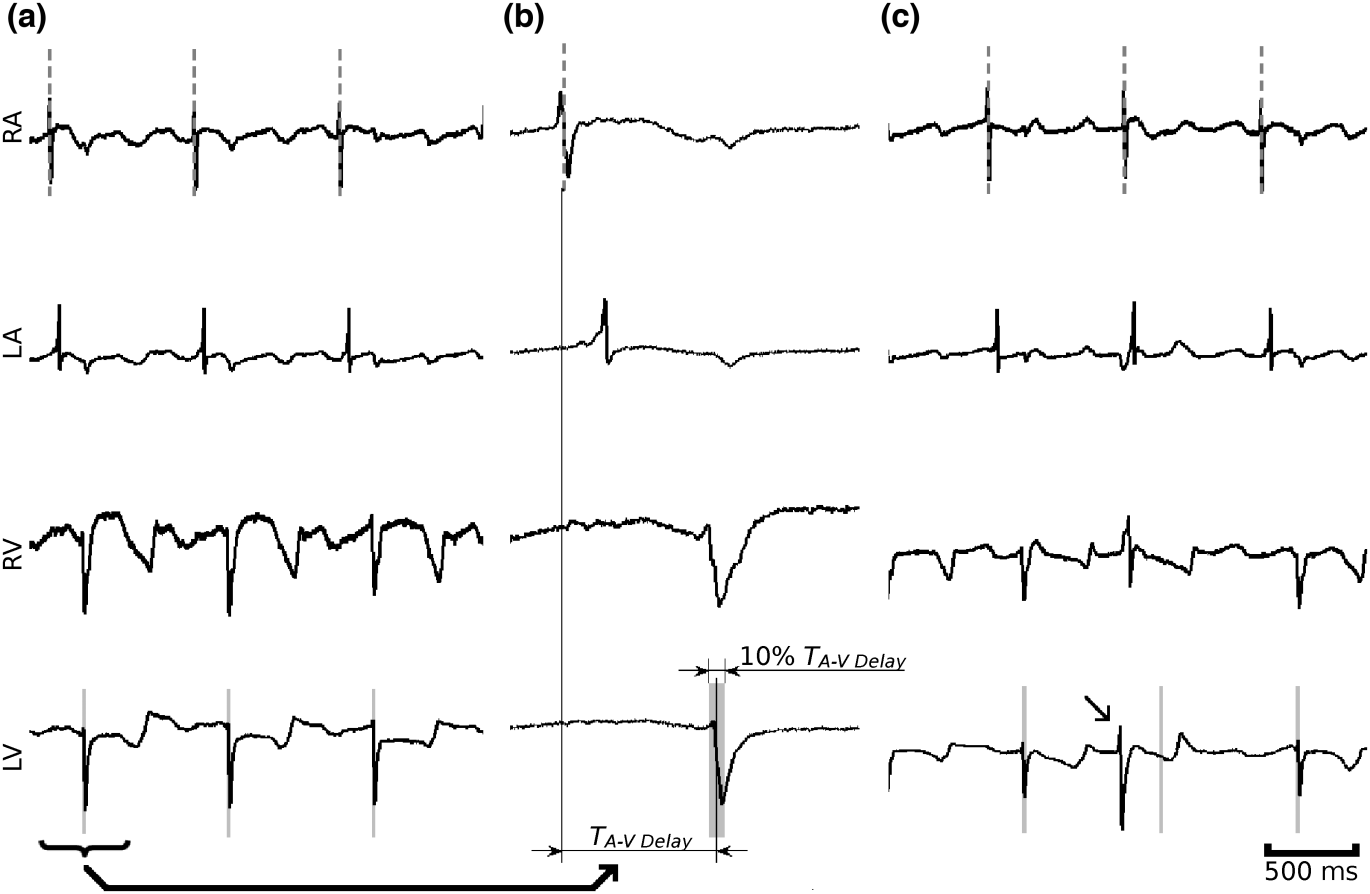
Unipolar electrogram signals from subepicardial hook electrodes. (a) Three normal sinus beats. Gray dashed lines indicate RA activation. (b) Expansion of the first beat in (a), showing the definition of *T*_A‐V delay_ and the time window of a sinus beat (gray shading) (c), a sequence of activations containing a ventricular ectopic beat (indicated by arrow). Ectopic beats occur outside of the expected time window. RA, right atrium

In all hearts, ventricular ectopic events were identified using electrograms from the subepicardial electrodes in the heart's four chambers as described in the Supplementary Methods. Briefly, as shown in Figure 2, we sought an abnormal delay (*T*_A‐V delay_) between RA and LV activation. In NW and LVW hearts, data epochs containing each ectopic event and at least one complete normal cardiac cycle prior to that irregular event were exported from the mapping datasets for further analysis. VT/VF and events consisting of multiple ectopic beats were counted as a single event and the origin of the first ectopic beat was investigated. The beginning of phase 1b is associated with impedance changes (Cinca et al., 1997). Because we did not measure impedance, events occurring before 15 min post‐occlusion were deemed to be in phase 1a and those occurring later in phase 1b (Coronel et al., 2002).

#### 2.4.2 Spatial origin of ectopic activations

Optical mapping data epochs were processed as previously described (Zhang et al., 2016). The majority of motion artifact in the optical *V*_m_ signals recorded from the *marked region* (defined as the epicardial region spanned by trackable markers, Figure 1c) was successfully suppressed. The location of the earliest activation within the marked region for each ectopic beat was identified by animating the optical mapping data. In cases in which the earliest activation was located near the edge of the marked region, we examined an *expanded mapping region* that extended from the edge of the marked region to the silhouette of the heart (Figure 1c). This region lacked trackable markers, but partial correction of motion artifact was possible using excitation ratiometry alone (Bachtel et al., 2011). In this region, depolarization could usually be reliably identified, expanding the region in which earliest activations could be identified, even though residual motion artifact typically obscured repolarization. The earliest electrical activation times recorded by the needle electrodes in the ventricular septum and LV posterior wall for each ectopic event were also identified.

Using the perfusion boundary identified by infusing hearts with fast green dye (Figure S1), the mapping regions were categorized into three zones: Normal Zone (NZ), Ischemic Border Zone (IBZ), and Ischemic Zone (IZ). The IBZ in the marked region was defined by the set of marker triangles that contained the perfusion boundary (approximate width of 7–14 mm; Figure 1c). In the ventricular septum, the IBZ was defined with a similar width, that is, three electrodes on each side of the perfusion boundary (approximate width of 10 mm; Figure 1d). This scheme provided an IBZ width similar to previously reported IBZ width in pig hearts (on the order of 8–12 mm) (Janse et al., 1979).

The spatial origin (focus) of an ectopic beat was identified as the electrical or optical recording site with the earliest activation time. The locations of ectopic foci were then categorized based on the perfusion zone in which they were located: NZ, IZ, or IBZ. We also noted the anatomic region in which foci were located: LV, Septal, or RV. Some ectopic activations had the same propagation pattern as that of a normal sinus beat but had no prior atrial activation or had markedly shorter atrioventricular delay. These activations were considered to have originated from upstream sites in the cardiac conduction system. The origin of some ectopic activations was deemed undetermined. The Supplementary Methods describe the situations in which this was the case.

#### 2.4.3 Measurement of action potential duration

During hypoxia, ATP‐sensitive potassium channels open, which abbreviates action potential duration (APD; Shaw & Rudy, 1997). APD can therefore be used as an approximate indicator of tissue oxygenation. We measured APDs at 5‐min intervals beginning at the time of occlusion. APD was computed using the time difference between peaks of the first and second temporal derivatives of each signal as in our previous publication (Zhang et al., 2016). For each animal, we obtained a single APD value for each perfusion zone by averaging APD over the triangles in that perfusion zone.

#### 2.4.4 Measurement of mechanical deformation

Mechanical deformation in the marked region was measured as in our previous publication (Zhang et al., 2016). Briefly, over the duration of each data epoch, strains for each triangle formed by epicardial markers were calculated using the end‐diastolic state of the normal cardiac beat as the reference state. This method assumes homogeneous strain within triangles; see the Supplementary Methods for validation in this experimental model. Principal strains (*S*_1_ < *S*_2_) were used to characterize mechanical deformation. During systole of normal contraction, the first principal strain, *S*_1_, is large and negative, while *S*_2_, the deformation in the orthogonal direction, is small and positive. With local loss of contractility, shortening is lost, and both *S*_1_ and *S*_2_ may become positive during systole as dyskinesia develops. See Figure 8b in our previous publication for an example (Zhang et al., 2016). Positive *S*_1_ (which implies positive *S*_2_) is therefore an indicator of mechanical stretch.

The maximum of mechanical stretch or shortening does not necessarily occur at the moment of end‐systole because it is the result of the interaction of cavity pressure, residual contractility in ischemic tissue, and mechanical tethering to normally contracting tissue. For each marker triangle, we identified *stretch magnitude* and *shortening magnitude*. Stretch magnitude is the maximum positive major strain *S*_2_ (when *S*_1_ > 0) during a cardiac cycle. When *S*_1_ ≤ 0 through the entire cardiac cycle, the stretch magnitude was 0. Shortening magnitude is the most negative *S*_1_ (when *S*_1_ < 0) during a cardiac cycle. When *S*_1_ ≥ 0 through the entire cardiac cycle, the shortening magnitude was 0.

Mechanical function during the early stages of ischemia changes over time (Allen & Orchard, 1987). In addition to characterizing the mechanical function of the normal beats preceding each ectopic activation, we computed both stretch and shortening magnitudes of normal cardiac cycles every 5 min in the first 20 min post‐occlusion (when change is most rapid) and every 15 min thereafter. For each animal, the stretch and shortening magnitudes in each perfusion zone were the values averaged over triangles in that perfusion zone.

#### 2.4.5 Statistical analysis

We used a two‐factor mixed model with interaction to compare ectopy rate (events per minute) between the two preparations (PREP: LVW or NW, between‐subjects factor), and ischemia phase (PHASE: 1a or 1b, within‐subjects factor). We used ectopy rate rather than event counts because phase 1a and 1b have different durations. Ectopy rate was not normally distributed across hearts, so we employed the aligned rank transformation (ART), which supports nonparametric two‐way mixed models (Wobbrock et al., 2011). We used the ART‐C method with Holm–Bonferroni correction to conduct post hoc comparisons (Elkin et al., 2021). These tests were carried out with the R language (v4.2.1; R Core Team) version of ARTool (v0.11.1). To compare ectopy in sham hearts with NW hearts, we used the nonparametric Wilcoxon rank‐sum test.

In analyzing post‐occlusion mechanical function, we tested both mechanical shortening and stretch magnitudes using linear mixed models with compound symmetry covariance structure. We evaluated the effects of occlusion time (TIME), perfusion zone (ZONE: NZ, IBZ, or IZ), and preparation (PREP: LVW or NW). TIME and ZONE were within‐subjects factors. Post‐occlusion APD shortening, ΔAPD, was tested using a similar linear mixed model. These tests were carried out using SPSS (v25; IBM Corp).

## 3 RESULTS

Descriptive data are given as *median* (*IQR*: *interquartile range*) or *mean ± SD*.

### 3.1 Post‐occlusion ectopic activity

**FIGURE 3:**
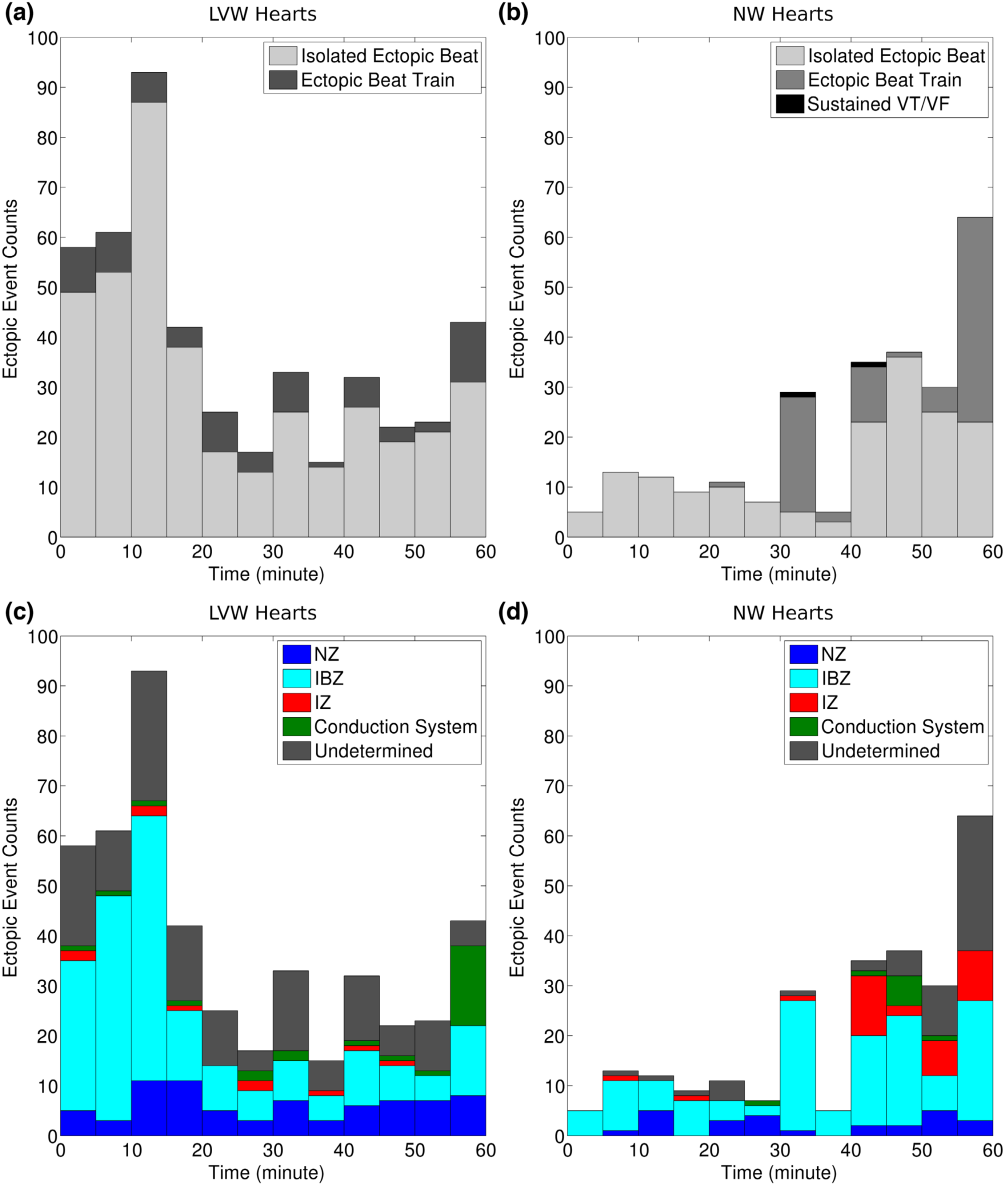
Classification of all post‐occlusion ectopic events in eight LVW and eight NW hearts. (a, b) Events grouped by arrhythmia type. (c, d) Events grouped by spatial origin. Statistical analysis by aligned‐rank transformation mixed model (see text for details). LVW, left ventricular working mode; NW, non‐working

Table 1 and Figure 3 show the overall post‐occlusion ectopic activity grouped by phase (1a or 1b) and spatial origin. The nonparametric mixed model had a significant interaction between PREP and PHASE (p = 0.005); the PREP main effect was also significant (*p* = 0.025), but PHASE was not (*p* = 0.10). The post hoc tests showed that ectopy rate differed significantly between LVW and NW hearts during phase 1a (*p* = 0.02), but not during phase 1b (*p* = 0.37). Ectopy rate did not differ significantly between phases 1a and 1b for either LVW or NW hearts (*p* = 0.37, *p* = 0.26, respectively). There was not a significant difference between the number of ectopic events in the NW and sham hearts (*p* = 0.24).

**TABLE 1:** Overall post‐occlusion ectopic activity (additional data on spatial origin are in Table S1).

	LVW median (interquartile range)	NW median (interquartile range)	Sham median (interquartile range)
Sample size	*N* = 8	*N* = 8	*N* = 3
Total ectopic events	51 (30–83)	19 (14–38)	11 (10–12)
1a ectopic events	19 (10–28)	2 (1–6)	5 (4–7)
1b ectopic events	27 (22–42)	16 (12–31)	7 (5–7)

Abbreviations: LVW, left ventricular working mode; NW, non‐working.

We examined ectopic events with foci registered in the epicardial marked region and extended mapping regions to determine if ectopic events preferentially arose from the IBZ. Under the null hypothesis that ectopic foci are uniformly distributed in the IBZ and non‐IBZ regions, we would observe the fraction of IBZ events to be similar to the relative area of the IBZ, which was 25% ± 4% and 26% ± 6% of the extended mapping region in the LVW and NW groups, respectively. Table 2 shows that this was not the case, with significantly more events in IBZ than non‐IBZ regions in both NW and LVW groups (*p* < 0.01, chi‐square test). This significance did not change even when all ectopic events with undetermined origin were assumed to arise from the non‐IBZ regions. These data, therefore, indicate that ectopic beats arose preferentially from the IBZ.

**TABLE 2:** Expected LV IBZ ectopic activity based on LV IBZ area and observed LV IBZ activity.

LV region	Average area percentage (%)	Expected IBZ ectopic events	Observed IBZ ectopic events
LVW			
LV IBZ	25	42.5	119
LV Non‐IBZ	75	127.5	51
NW			
LV IBZ	26	38.22	95
LV Non‐IBZ	74	108.78	52

Abbreviations: IBZ, ischemic border zone; LV, left ventricular; LVW, left ventricular working mode.

### 3.2 Post‐occlusion mechanical function

**FIGURE 4:**
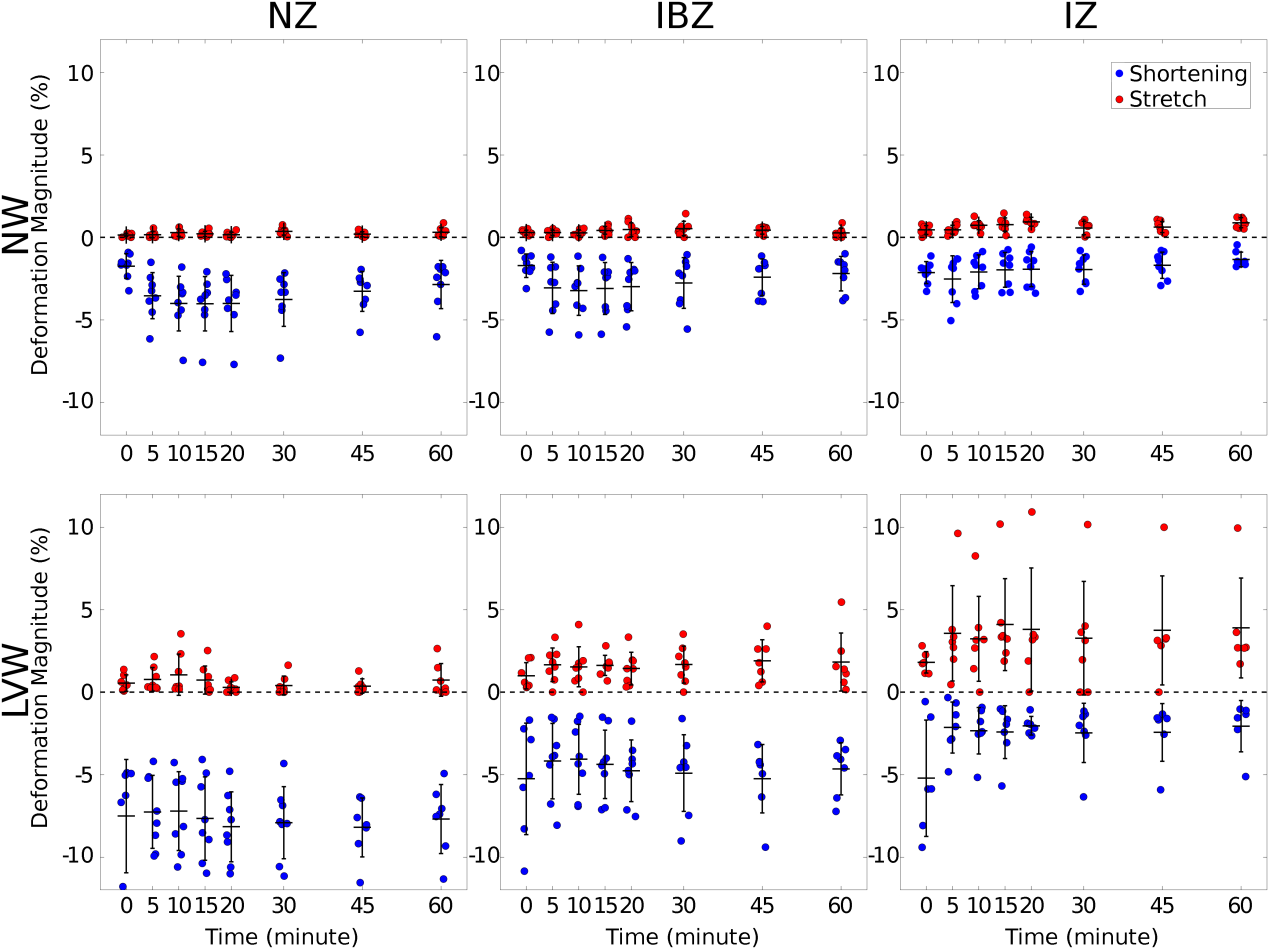
Post‐occlusion deformation magnitudes by preparation (LVW or NW) and perfusion zone (NZ, IBZ, IZ). Stretch magnitudes are red and shortening magnitudes are blue. Black bars show mean and SD. There were eight LVW and eight NB hearts. Each data point is the average of all triangles within the respective prefusion zone. Statistical analysis by linear mixed model (see text for details). IBZ, ischemic border zone; IZ, ischemic zone; LVW, left ventricular working mode; NW, non‐working; NZ, normal zone

Figure 4 shows the shortening and stretch magnitudes measured periodically during occlusion. Table 3 shows the significance of the factors and interactions.

**TABLE 3:** Statistical results of post‐occlusion mechanical function and APD shortening.

Factors	Significance		
	Shortening	Stretch	ΔAPD
TIME	*p* = 0.004	*p* = 0.076	*p* < 0.001
ZONE	*p* < 0.001	*p* < 0.001	*p* < 0.001
PREP	*p* = 0.013	*p* = 0.003	*p* = 0.097
TIME × ZONE	*p* < 0.001	*p* = 0.595	*p* < 0.001
ZONE × PREP	*p* < 0.001	*p* < 0.001	*p* < 0.001
PREP × TIME	*p* < 0.001	*p* = 0.317	*p* = 0.039
TIME × ZONE × PREP	*p* = 0.887	*p* = 0.938	*p* = 0.118

Abbreviation: APD, action potential duration.

Blebbistatin reduced shortening in the NW hearts, more so in the NZ and IBZ than the IZ (significant ZONE × PREP). However, nonzero shortening magnitudes were nevertheless present in NW hearts, showing that blebbistatin did not eliminate contraction. The significance of TIME × PREP indicates that the shortening magnitude changed throughout the experiment, more so in the NW hearts. We tested a contrast comparing shortening magnitude at TIME 0 with shortening magnitude averaged over all other TIMEs in the NW hearts. Shortening magnitude at TIME 0 was significantly less negative (*p* < 0.01), indicating that contraction at least partially recovered from blebbistatin treatment starting 5 min after the onset of optical mapping.

Mechanical stretch was pronounced in IBZ and IZ of LVW hearts. Contrasts comparing stretch magnitude in the IZ and IBZ of NW hearts with the NZ of LVW hearts were not significant (*p* = 0.86 and *p* = 0.59, respectively), indicating that stretch in the ischemic zones of the NW hearts was not different from stretch in the normally contracting regions of the beating LVW hearts. On the other hand, contrasts comparing stretch magnitude in the IZ and IBZ of LVW hearts with the NZ of LVW hearts were significant (*p* < 0.01 for both), indicating that in these beating hearts, stretch in the ischemic zones was greater than the stretch in the normally contracting regions.

### 3.3 Post‐occlusion APD shortening

We use APD shortening of sinus beats as a surrogate for hypoxia. We adjusted the perfusion flow rate in NW hearts so that the dissolved oxygen content of the coronary effluent was approximately the same as that in LVW hearts (7.9 ± 0.6 mg/L vs. 7.6 ± 0.8 mg/L, *p* = 0.41). Nevertheless, APD immediately before coronary occlusion (time 0) was significantly less in LVW than NW hearts (285 ± 27 ms vs. 339 ± 44 ms, *p* < 0.01), suggesting that baseline global tissue oxygenation was less in LVW hearts, probably because of their higher mechanical load (Wengrowski et al., 2014). It is worth noting that sinus cycle length at time 0 did not differ significantly between preparations (658 ± 94 ms vs. 611 ± 80 ms, *p* = 0.34), indicating that the APD difference was not simply attributable to a difference in activation rate.

**FIGURE 5:**
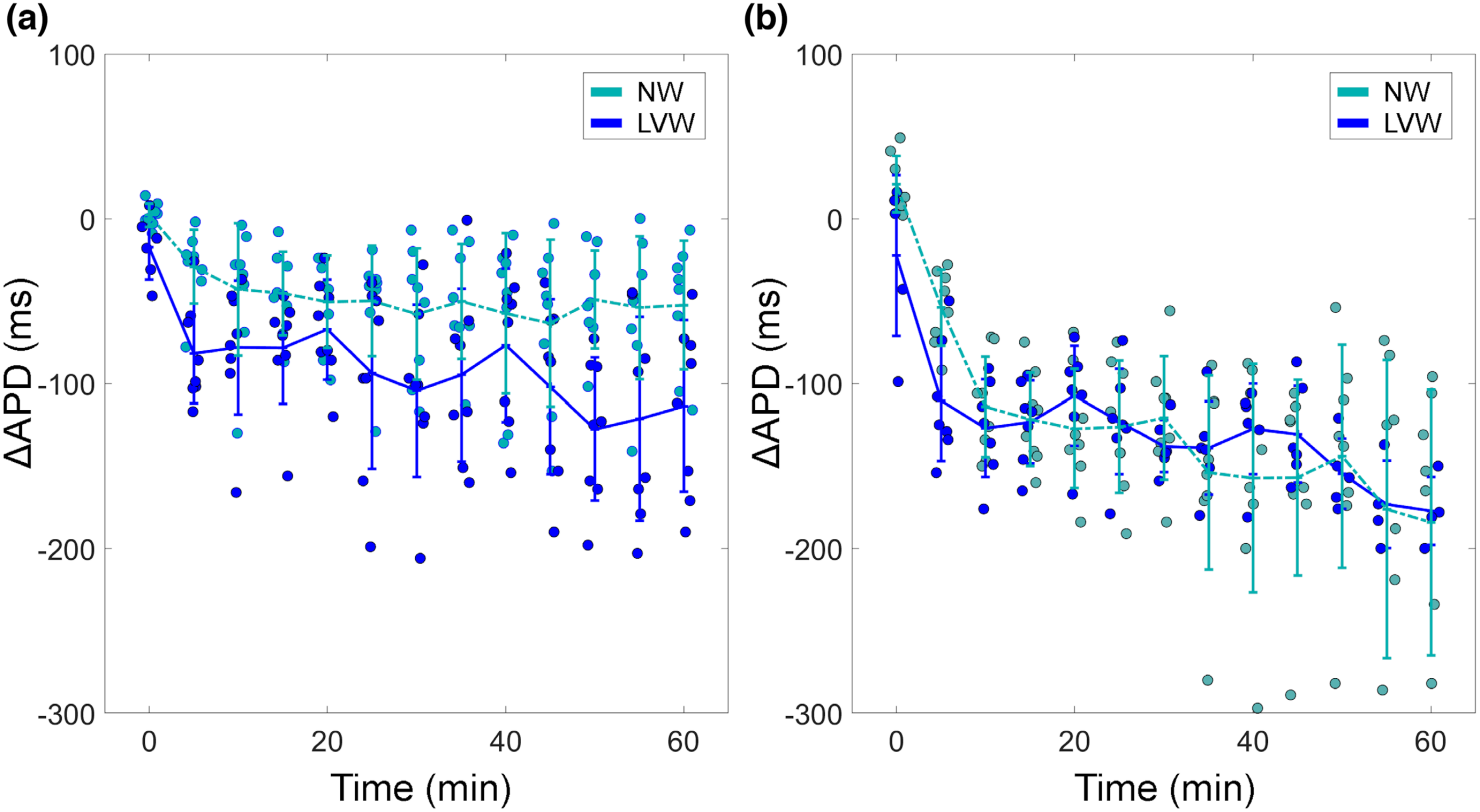
Post‐occlusion APD shortening. (a) ΔAPD_IBZ_ is the average APD in normal zone triangles subtracted from the average APD in IBZ triangles. It differs in LVW (dark blue) and NW (light blue) hearts (*p* = 0.01). (b) ΔAPD_IZ_ (computed in the same way as ΔAPD_IBZ_) does not differ in LVW (dark blue) and NW (light blue) hearts (*p* = 0.52). There were eight LVW and eight NW hearts. Statistical analysis by linear mixed model (see text for details). APD, action potential duration; IBZ, ischemic border zone; LVW, left ventricular working mode; NW, non‐working

We defined ΔAPD as the difference in APD in the IZ or IBZ relative to APD in the NZ of the same heart, i.e., ΔAPD_IBZ/IZ_ = APD_IBZ/IZ_ − APD_NZ_. Table 3 shows the significance of factors and interactions from the linear mixed model. We tested contrasts comparing ΔAPD_IZ_ and ΔAPD_IBZ_ of the LVW hearts with their counterparts in the NW hearts. The ΔAPD_IBZ_ contrast was significant (Figure 5a, *p* = 0.01) but the ΔAPD_IZ_ contrast was not (Figure 5b, *p* = 0.52), suggesting that, under partial perfusion conditions, loaded ventricular tissue is more susceptible to hypoxia than unloaded tissue, while under complete ischemic conditions, ventricular tissue suffers effects of hypoxia whether mechanical load is present or not.

### 3.4 Mechanical stretch at ectopic foci in LVW hearts

To investigate the presence of mechanical stretch at ectopic focal sites, we analyzed ectopic events from LVW hearts that occurred within the marked region, where both electrical activation and mechanical deformation data were available. Sixty‐nine of the 464 ectopic events in the LVW group met this criterion.

**FIGURE 6:**
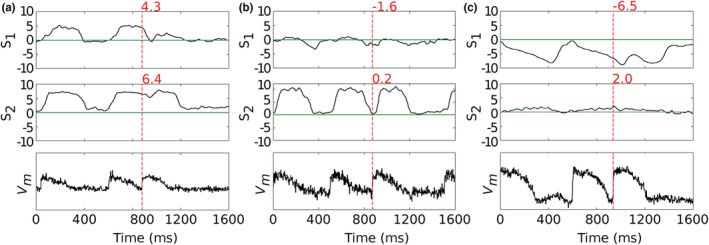
Examples of principal strains *S*_1_ and *S*_2_ (in %) and *V*_m_ at ectopic focal sites. Red dashed lines indicate the moment of depolarization (as indicated by maximum *dV*_m_/*dt*). Values in red are the strains at depolarization time. (a) The ectopic focal site was stretched (i.e., *S*_1_ > *S*_2_ > 0) at depolarization time. (b) The ectopic focal site was not stretched at depolarization time but had been stretched during the prior cardiac cycle. (c) The ectopic focal site was never stretched during the previous cardiac cycle. See additional examples in Figure S4.

**FIGURE 7:**
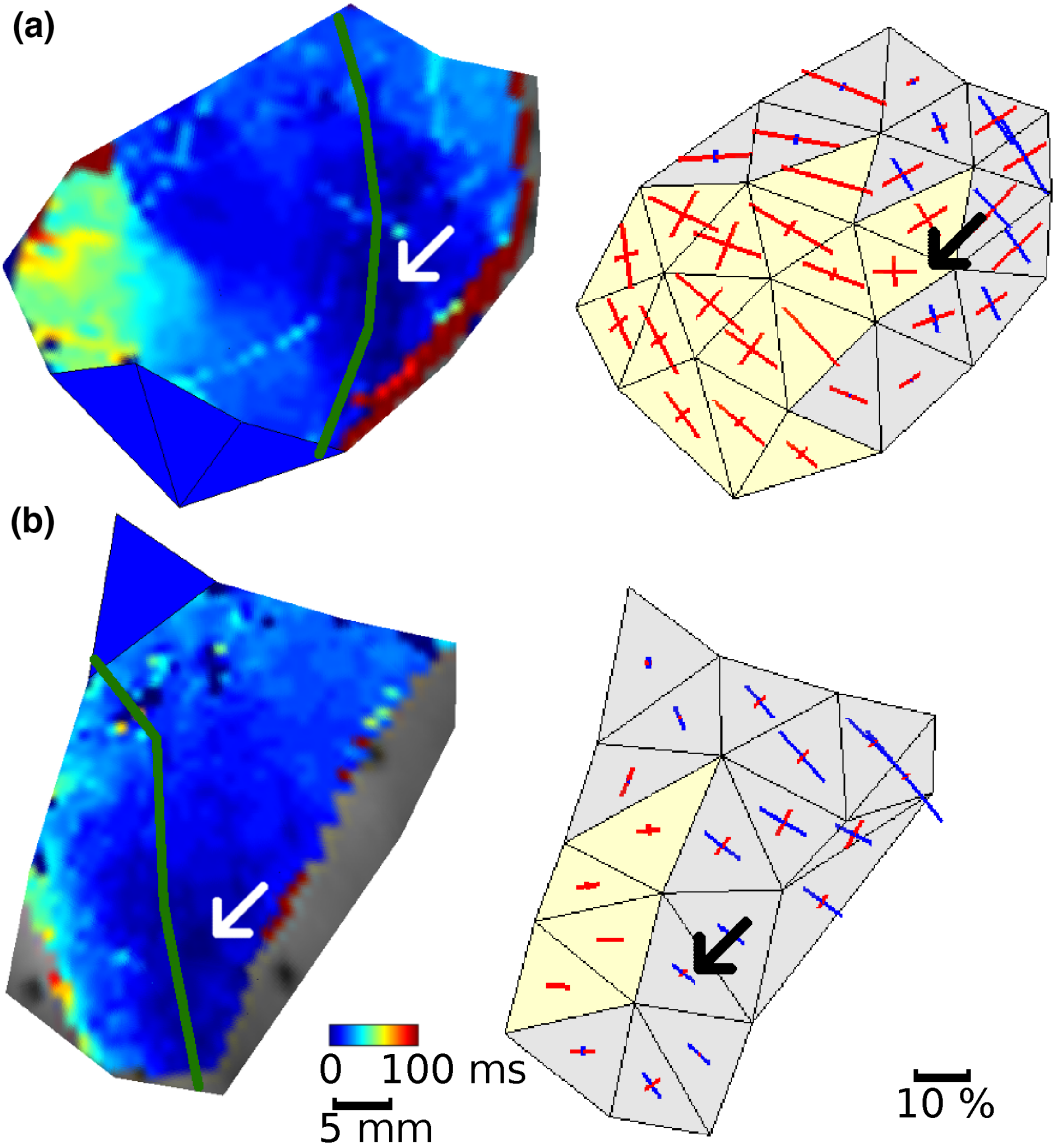
Isochronal activation maps of ectopic activation (left panels) and the distribution of mechanical strains at the moment of ectopic depolarization (right panels) in the marked region. Ectopic foci are indicated by arrows. *V*_m_ could not be reconstructed in the solid blue triangles in the left panels. Dark green lines indicate the perfusion boundary. Blue and red bars represent negative and positive principal strain, respectively. Bars are oriented in the respective principal direction. Triangles in which both principal strains are positive (i.e., stretched) are yellow. (a) The ectopic focal site is stretched at the moment of depolarization. (b) The ectopic focal site is not stretched at the moment of depolarization.

Using positive stain in both principal directions to indicate mechanical stretch, we categorized these 69 ectopic events into three groups: *I*, The ectopic focal site was stretched at the moment of ectopic depolarization, for example, Figures 6a and 7a. There were four such events, all from the IBZ and occurring during phase 1b. This was 6% of events (95% confidence interval: 2%–14%). *II*, The ectopic focal site was stretched at some time during the cardiac cycle immediately preceding ectopic activation, but not at the moment of depolarization, for example, Figure 6b. Five events met this criterion, two from the IBZ, three from the NZ, one during phase 1a, and four during phase 1b (7% of total events, 95% confidence interval 3%–16%). *III*, The ectopic focal site was never stretched during the last cardiac cycle preceding ectopic activation, for example, Figures 6c and 7b. The remaining 60 events were in this group (39 IBZ, 21 NZ; 27 during 1a, 33 during 1b).

We compared ectopic focal site APDs with the APDs of nearby sites (<3 cm apart) in the NZ. For events that originated in the IBZ, focal site APDs were significantly shorter than NZ APDs (*p* < 0.01, paired t‐test), suggesting local hypoxic conditions (Shaw & Rudy, 1997). For events that originated in the NZ, focal site APDs did not significantly differ from the APDs in nearby NZ sites (*p* = 0.78, paired *t*‐test).

We compared the stretch at the ectopic focal sites with concurrent stretch in all non‐IZ triangles in the marked region. None of the ectopic focal sites were in the most‐stretched triangle at the moment of ectopic depolarization and only one ectopic focal site had the greatest stretch magnitude through the prior cardiac cycle. The average distance between ectopic focal sites and the most‐stretched non‐IZ triangles was 16 ± 7 mm (triangle‐centroid‐to‐triangle‐centroid end‐diastolic distance; see scale bar in Figure 7 for spatial reference).

We also examined whether deformation was more heterogeneous at ectopic sites than other sites. To quantify heterogeneity, for each ectopic event, we identified the triangle containing its focus. The differences in deformation magnitude between the ectopic triangle and its three (or two, if on edge) neighboring triangles were found and the variance of these differences was calculated. Three ectopic sites had only one neighbor and were excluded, leaving 66 ectopic events. For each event, we randomly selected another triangle from the same perfusion zone as a non‐ectopic control and repeated this calculation. Paired *t*‐tests showed there was no significant difference in heterogeneity of either stretch or shortening magnitude between the ectopic and control sites (Figure S4).

**FIGURE 8:**
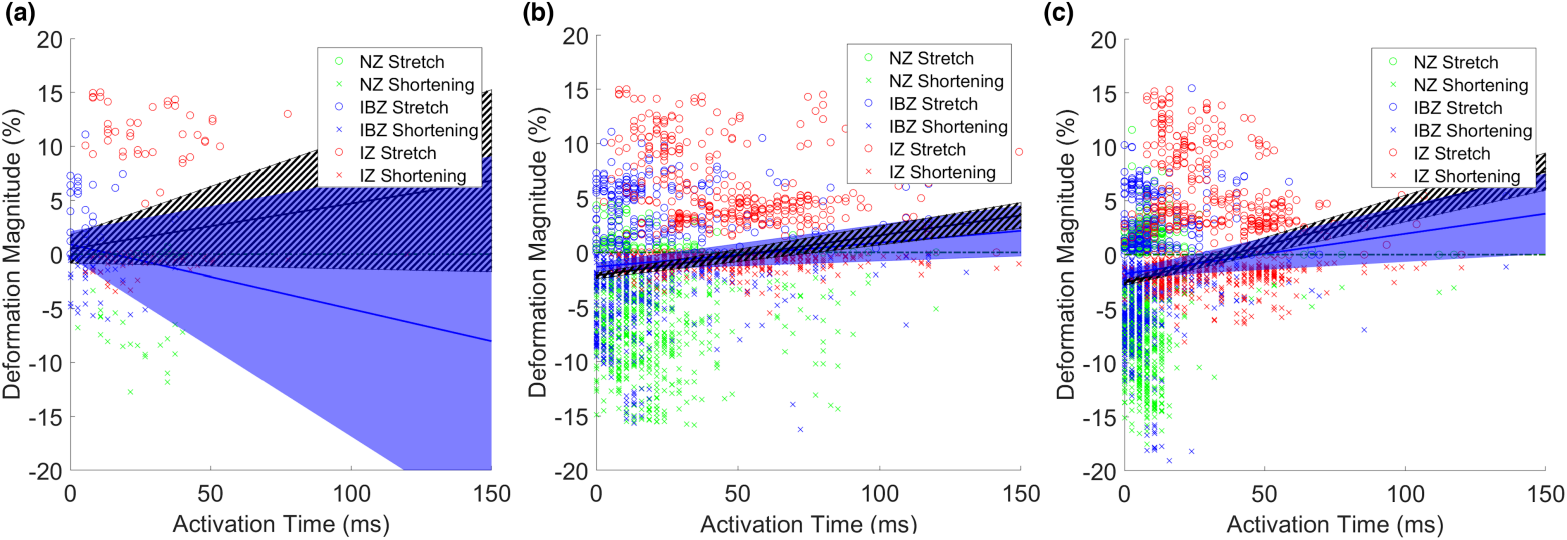
Deformation magnitude versus activation time for marker triangles in LVW hearts in which both mechanical and electrical data were available. Activation times are measured at the center of marker triangles and are relative to the earliest activation for that beat. There are two data points per activation: one each for stretch and shortening magnitude measured over the cardiac cycle preceding activation. Plot markers are color‐coded by perfusion zone. The blue lines and shadows are regression lines and 95% CI for triangles in the IBZ only. Black lines/hatched regions show the regression and 95% CI for all triangles. (a) Data from only the four group I ectopic beats at which the earliest activated sites were stretched at the time of activation. (b) Data from all 69 ectopic beats with early sites within the marked region. (c) Data from 72 non‐ectopic beats (9 beats in each of 8 animals) during the post‐occlusion period. CI, confidence interval; LVW, left ventricular working mode

Figure 8 shows the relationship between a marker triangle's activation time (measured at its centroid) and its two deformation magnitudes (stretch and shortening) measured during the cardiac cycle preceding activation. Details for the regressions shown in the figure are in Table S2. Figure 8a includes data only from the four group *I* beats (in which the early site was stretched at the time of activation). Figure 8b includes data from all 69 ectopic beats, and Figure 8c includes data from a similar number of non‐ectopic beats. Data from all ectopic beats resemble data from the non‐ectopic beats in that the regressions have significantly negative intercepts and significantly positive slopes. In contrast, the blue regression in Figure 8a, which was fitted only to IBZ triangles, differs in that its intercept is positive and its slope is negative. Although not significant, this later pattern is what we would expect of activation waves originating from stretched myocardium and propagating away into less stretched or contracting myocardium.

## 4 DISCUSSION

### 4.1 Major findings

It is well known that following coronary occlusion, myocardial force production in the ischemic region decreases within a few minutes (Allen & Orchard, 1987). Preserved contraction in nonischemic regions working against intraventricular pressure causes the noncontracting parts of the ventricular wall to become dyskinetic and bulge outward during systole (Tennant & Wiggers, 1935), stretching myocardium. It is also well known that stretch can potentiate depolarizing current and trigger focal electrical activation (Franz et al., 1992; Zeng et al., 2000). A number of investigators have therefore proposed that stretch arising from dyskinesia could trigger the ectopic beats that lead to arrhythmias during the early stages of acute regional ischemia (Coronel et al., 2002; Franz et al., 1992; Jie et al., 2010; Parker et al., 2004). If this is indeed the case, sites of ectopic activation would be expected to co‐locate with sites undergoing stretch.

We modeled acute regional ischemia in an isolated LVW preparation in which the pulmonary veins and aorta were cannulated and connected to hydrostatic pressure loads. We recently introduced an optical mapping method that makes it possible to simultaneously map epicardial strain distributions and electrical activation in such a beating preparation (Zhang et al., 2016). Consistent with previous studies (Coronel et al., 2002) in our experiments, ectopic activation was more likely during phase 1a of acute regional ischemia in mechanically loaded hearts in which ventricular dyskinesia was present than in unloaded hearts with suppressed contraction. Despite this, our major finding is that ectopic activations during both phase 1a and phase 1b only occasionally co‐locate with sites that are undergoing epicardial stretch, suggesting that focal beats triggered by stretch that is evident on the epicardium are not a major source of ventricular ectopy during acute regional ischemia.

### 4.2 Ventricular ectopy in working and NW hearts

In the LVW preparation, we found that following coronary occlusion, the onset of stretch in the IZ and IBZ was rapid (<5 min) with stretch magnitude in both the IZ and IBZ significantly exceeding stretch magnitude in the NZ. This is consistent with previous findings on the gradients of post‐occlusion epicardial strains (Van Leuven et al., 1994).

In the NW hearts, which were treated with blebbistatin to suppress dyskinesia, it is important to note that mechanical contraction was reduced, but not eliminated. This is most likely because blebbistatin is inactivated by light of the wavelength that was used for optical mapping (Sakamoto et al., 2005). In our experiments, the shortening magnitude in NZ of NW hearts was <2% at the beginning of optical mapping but then increased to ~4% upon light exposure (Figure 4). This initial increase in shortening magnitude was not observed in LVW hearts, which were not treated with blebbistatin. However, despite the partial recovery of contraction, because of the absence of intraventricular pressure, stretch magnitude in the IBZ and IZ was very small and indistinguishable from the stretch magnitude in the NZ of the contracting LVW hearts, indicating that the residual contraction in the NZ of the NW hearts was insufficient to elicit stretch in the IZ and IBZ regions of the NW hearts above the level of the normally contracting regions of the beating LVW hearts.

During phase 1a of acute regional ischemia (the first 15 min post‐occlusion), there were significantly more ectopic activations in the loaded and contracting LVW hearts (19 [IQR: 10–28] beats/heart) than in the unloaded NW hearts (2 [IQR:1–6] beats/heart). Indeed, the number of ectopic beats in NW hearts could not be distinguished from the level of ectopy in the sham hearts, which were prepared identically to the NW hearts, but did not undergo coronary occlusion. This suggests that when stretch was absent, regional ischemia did not increase ectopy above the preparation's background level. These data support the broad hypothesis that mechanical loading is a factor in ectopy during phase 1a.

However, an alternative explanation for the difference in phase 1a ectopy between the LVW and NW hearts is that rather than being due to stretch‐induced depolarizations, it was due to the higher oxygen consumption of beating hearts (Wengrowski et al., 2014). This could lead to a mismatch in myocardial oxygenation between the LVW and NW groups, and consequently, a confounding mismatch in electrophysiological properties (Garrott et al., 2017). To compensate for this, we adjusted the coronary flow rate in the Langendorff‐perfused NW hearts so that oxygen content in the coronary effluent matched the level measured in the LVW hearts. Nevertheless, APD, which shortens during hypoxia, was abbreviated just before coronary occlusion in the LVW hearts relative to the NW hearts. This suggests a more pronounced mismatch between oxygen supply and demand in the LVW hearts, which could have contributed to elevated ectopy.

In contrast with phase 1a, during phase 1b (15–60 min post‐occlusion), the number of ectopic beats did not significantly differ between the LVW and NW groups (27 [IQR: 22–42] vs. 16 [IQR:12–31]). This is inconsistent with Coronel et al. (2002) who found less phase 1b ectopy in unloaded compared to loaded hearts. In Figure 3, ectopy rate appears to decrease going from phase 1a to 1b in LVW hearts but to increase in NW hearts. Neither change alone is significant in post hoc analysis, so both together probably account for the lack of a significant difference in phase 1b ectopy rate between LVW and NW hearts. An increase in ectopy rate between phase 1a and 1b is unexpected (Smith et al., 1995). A possible explanation for this change in our study is that it resulted from blebbistatin phototoxicity. Blebbistatin has lesser side effects on cardiac electrophysiology than alternative agents such as 2,3‐butanedione monoxime or cytochalasin‐D (Li & Nattel, 2007). However, it has been reported that it is unstable under exposure to blue light and phototoxic after about 10–20 min, probably due to the products of photodegradation (Kolega, 2004; Mikulich et al., 2012). The possible increase in ectopy during phase 1b in the blebbistatin‐treated NW hearts may therefore be due to progressive blebbistatin phototoxicity, particularly in the poorly perfused IZ and IBZ where photoproducts could accumulate.

In both NW and LVW hearts, focal beats arose from the IBZ with frequency significantly out of proportion with the epicardial area occupied by the IBZ (Table 2). This was expected: While the IZ underwent more stretch than the IBZ (Figure 4), and any other ectopy‐promoting factors were also probably more pronounced in the IZ than IBZ, myocardium in the IZ is too electrically depressed, probably from potassium accumulation, to generate ectopic beats (Jie et al., 2010; Kléber, 1984; Shaw & Rudy, 1997; Van Leuven et al., 1994).

### 4.3 Mapping of ectopic foci and relation to stretch

To identify co‐location between dyskinetic stretch and ectopic activation, we used our newly developed electromechanical optical mapping system (Zhang et al., 2016) to simultaneously image strain distributions and electrical activation across the anterior ventricular epicardium. Mechanosensitive channel opening is believed to be very rapid (Peyronnet et al., 2016). Previous findings in isolated ventricular cells showed no noticeable delay between the application of stretch and the activation of stretch‐induced current; furthermore, the current did not sustain after the removal of stretch (Zeng et al., 2000). In whole heart studies, mechanical stretch resulted in depolarization, but with a time delay of up to 400 ms, depending on the strength of stimulation (Stacy et al., 1992), possibly because of the time required for tension to be mechanically transmitted to channels and for the activation threshold to be reached. We, therefore, sought to determine whether ectopic foci arose from sites that had undergone stretch from the moment of ectopic depolarization ranging back through the prior cardiac cycle.

We were able to document 69 ectopic events that initiated from the LV‐marked region in the contracting LVW hearts. This is the area in which data on both electrical activation and mechanical deformation were available. Only four of those events were co‐located with stretch at the time of ectopic electrical activation, and only five more were co‐located with stretch at any time during the previous cardiac cycle. We computed the distance between ectopic foci and the non‐IZ triangle that underwent the most stretch. In only one event was the ectopic site in the most‐stretched triangle. Overall, ectopic sites were an average of 16 ± 7 mm from the most‐stretched non‐IZ site in the marked region. In addition, we explored the relationship between mechanical deformation during the cardiac cycle preceding each ectopic activation and electrical activation time (Figure 8). If stretch was driving ectopy, we would expect to see net‐positive deformation at the earliest‐activating triangles. As the wave propagates away from the early site, we would expect to see negative deformation becoming more prominent—at least in the NZ and IBZ, which are still able to contract. This is the general pattern seen in the IBZ triangles of the four group *I* beats (in which the early site was stretched at the time of activation; Figure 8a, blue regression). The regression, while not significant, has the expected positive intercept and negative slope. In contrast, this pattern is not evident when data from all 69 ectopic beats are considered (Figure 8b). In the regression for IBZ data only (blue regression), the intercept is negative and the slope is positive. Data from the IBZ of a similar number of non‐ectopic beats are strikingly similar (Figure 8c, blue regression). When regression lines are fitted to data from all three perfusion zones (Figure 8a–c, hatched regressions), the slopes are positive, probably because the NZ, which is less stretched, is more excitable and activates early, while the IZ, which is more stretched, is less excitable and activates late. We further explored the potential relationship between ectopic activity and local mechanical heterogeneity at the ectopic foci during the cardiac cycle preceding each ectopic event (Figure S4). Our data show that mechanical heterogeneity at ectopic foci is not significantly higher than at non‐ectopic sites within the same perfusion zone. Together, these data suggest that local stretch leading to depolarizing current and electrical activation was *not* the primary cause of increased ectopic activation in the LVW hearts relative to the NW hearts.

Instead, the increased ectopy in the LVW hearts may have been due to a more ischemic IBZ milieu rather than to mechanosensitivity. We adjusted coronary flow rate in the NW hearts to provide a global oxygen supply–demand relationship similar to the LVW hearts (as indicated by the similarity between the oxygen content of the coronary effluent in both preparations). Nevertheless, before regional ischemia, APD was shorter in the LVW than NW hearts, suggesting possibly more hypoxic global conditions. Also, the difference in APD_NZ_ − APD_IBZ_ in the LVW compared to NW hearts (Figure 5a) indicates that local hypoxia in the IBZ was more pronounced in the LVW than NW hearts. This was likely due to the metabolic demand of mechanical contraction in the poorly perfused IBZ.

### 4.4 Limitations

An important limitation of the present study is that the region in which both electrical and mechanical data were available was limited to the portion of the epicardium spanned by markers that remained within the camera frame for the entire recording and whose motion was primarily parallel the camera plane (Zhang et al., 2016). Although additional mapping data were available from needle electrodes and the “expanded mapping region,” which consisted of partially motion‐corrected optical signals from outside of the marked region, these data were used primarily to document that apparent ectopic sites within the marked region were global early sites. Our study, therefore, assumes that the sample of ectopic sites that occurred within the marked region is reflective of the rest of the left ventricle.

Our electromechanical optical mapping system is limited to epicardial data. In previous reports, ventricular deformation varied across the wall, with the endocardium generally undergoing greater deformation than the epicardium in both ischemic and perfused conditions (Fenton et al., 1978; Villarreal et al., 1991). Thus, it is possible that ectopic waves could have originated from stretched intramural tissue. If such a wave broke through and was detected on the epicardium at an unstretched site, co‐location between ectopy and stretch would not be registered. In our study, infarcts were transmural with the perfusion boundary perpendicular to the surface of the heart (Figure S2), which is consistent with previous publications (Munz et al., 2011). This reduces the likelihood that activations originating from one perfusion zone within the wall would be registered in a different zone upon reaching the epicardium.

With our electromechanical mapping system, *V*_m_ spatial resolution is near pixel‐level, but strain resolution depends on marker spacing, which was ~8 mm in this study. In our previous study in nonischemic isolated swine hearts, this spacing was sufficient for strain to be constant within each triangle. In fact, even doubling the spacing provided similar results (Zhang et al., 2016). In the presence of regional ischemia, it is possible that strain could vary within IBZ triangles. Therefore, we carried out experiments on three additional hearts to test triangles located in the IBZ for differences in strain homogeneity between baseline conditions and three ischemic time points (see Supplementary Methods for details). We did not find a significant difference (Figure S2), indicating our strain measurements were sufficiently resolved.

In the LVW hearts, the perfusion system's afterload pressure was set to 50 ± 5 mmHg, which is consistent with pressure used in previous studies (Coronel et al., 2002), but lower than the arterial blood pressure of conscious pigs. We chose this pressure because crystalloid perfusate carries substantially less oxygen than blood and higher afterload pressure requires increased oxygen consumption (Wengrowski et al., 2014). Insufficient oxygen supply to the working hearts could potentially introduce confounding factors from global hypoxia (Garrott et al., 2017). In pilot studies, we found that increasing the afterload pressure above 60 mmHg nearly halved the oxygen content in the coronary effluent (from 7.6 to 4.3 mg/L). Systolic bulging occurs primarily during the isovolumic phase of contraction when the ventricle is not exposed to the afterload pressure (Akaishi et al., 1986), so we do not believe this was a major factor in this study. However, it is possible that lower afterload pressure altered the time course of stretch in the IZ and IBZ and may have reduced stretch magnitudes.

Mechanotransduction in the heart may be driven by strain, stress, or a combination of both. In the present study, we imaged strain; regional stress cannot be monitored with our current instrumentation. Because the stress–strain relationship is complex and nonlinear, spatial stress distributions may differ from strain distributions, which could affect co‐localization of mechanical stimuli with ectopy if stress is the primary driver. However, evidence from a number of studies suggests that strain rather than stress is the more likely stimulus for mechanically induced depolarization (Quinn & Kohl, 2021).

We used APD shortening as an indirect indicator of hypoxia. NADH fluorescence is an alternative and more direct reporter of metabolism (Wengrowski et al., 2014), but its fluorescence is in the excitation band of di‐4 and would interfere with our *V*_m_ imaging. The effects of hypoxia on APD could have been confounded with the effects of stretch on APD. A number of studies have indicated that in isolated hearts, while stretch shortens APD when measured early in repolarization, it prolongs APD when measured late in repolarization, as it was in the present study (Youm et al., 2005). Therefore, in the LVW IBZ, APD prolongation due to stretch could have caused shortening due to hypoxia to be underestimated. Even if this was the case, it would only amplify our finding that in this study, LVW IBZ was more hypoxic than NW IBZ. It is worth noting that the experiment was carried out in room air and the IZ/IBZ epicardium was exposed to atmospheric oxygen. However, oxygenation by this route can only reach a thin layer of cardiac tissue (<200 μm; Radisic et al., 2006) and it did not reverse hypoxia in IZ/IBZ as significant APD shortening was present.

### 4.5 Conclusion

Our data indicate that while ectopy is more frequent during acute regional ischemia in isolated, mechanically loaded, contracting hearts than in unloaded hearts with suppressed contraction, the ectopic beats generally do not co‐locate with stretched epicardial ventricular myocardium. This suggests that acute ventricular dyskinesia producing wall stretch resulting in local depolarization is not the primary source of ectopy during this period.

## AUTHOR CONTRIBUTIONS

Hanyu Zhang and Jack M. Rogers designed experiments, conducted experiments, wrote, and edited the manuscript. Han Yu and Gregory P. Walcott conducted experiments and edited the manuscript.

## FUNDING INFORMATION

This work was supported by the National Institutes of Health (R01‐HL115108 to J.M.R.); and the American Heart Association (16PRE30980044 to H.Z.).

## CONFLICT OF INTEREST

The authors declares that there is no conflict of interest.

## DISCLAIMER

The manuscript is not submitted elsewhere or under consideration for publication. All authors have read and agreed with the submission of manuscript.

## Supporting information


Appendix S1
