# Supplementary material for: Ectopic foci do not co‐locate with ventricular epicardial stretch during early acute regional ischemia in isolated pig hearts
Source: Physiol Rep. 2022 Oct 18;10(20):e15492. doi: 10.14814/phy2.15492 (PMC9579492; doi:10.14814/phy2.15492)
Supplement: Supplementary file 1 — Appendix S1 [file PHY2-10-e15492-s001.docx]

# Supporting Material

# Ectopic Foci Do Not Co-Locate with Ventricular Epicardial Stretch during Early Acute Regional Ischemia in Isolated Pig Hearts

*Hanyu Zhang Ph.D.*^1^, *Han Yu M.S.*^1^*, Gregory P. Walcott M.D.*^2^*, Jack M Rogers Ph.D.*^1*^

^1^Department of Biomedical Engineering ^2^Department of Medicine

University of Alabama at Birmingham, Birmingham, Alabama, USA

^*^Correspondence: jrogers@uab.edu

# Supplementary Tables

Supplementary Table 1. Spatial origin of ectopic events

|  | LVW  MEDIAN (interquartile range) | NW  MEDIAN (interquartile range) | SHAM  MEDIAN (interquartile range) |
| --- | --- | --- | --- |
| Sample Size | N=8 | N=8 | N=3 |
| Total Ectopic Events | 51 (30-83) | 19 (14-38) | 11 (10-12) |
| 1a Ectopic Events | 19 (10-28) | 2 (1-6) | 5 (4-7) |
| 1b Ectopic Events | 27 (22-42) | 16 (12-31) | 7 (5-7) |
| Spatial Origin |  |  |  |
| IBZ | 19 (8-35) | 9 (3-23) |  |
| LV IBZ | 7 (3-15) | 4 (1-19) |  |
| RV IBZ | 4 (2-8) | 0 (0-1) |  |
| Septal IBZ | 3 (1-7) | 0 (0-6) |  |
| NZ | 8 (4-14) | 2 (0-3) |  |
| LV NZ | 5 (3-7) | 1 (0-3) |  |
| RV NZ | 1 (0-1) | 0 (0-0) |  |
| Septal NZ | 0 (0-2) | 0 (0-0) |  |
| IZ | 1 (0-2) | 1 (0-4) |  |
| LV IZ | 0 (0-0) | 1 (0-4) |  |
| RV IZ | 0 (0-0) | 0 |  |
| Septal IZ | 1 (0-1) | 0 |  |
| Conduction System | 0 (0-1) | 0 (0-1) |  |
| Undetermined | 19 (10-25) | 2 (0-7) |  |

Supplementary Table 2. Regression analysis for activation time-deformation magnitude relationship

| VPBs | Site | R^2^ | Parameter | | 95% CI | *p* |
| --- | --- | --- | --- | --- | --- | --- |
| VPBs from mapping region | All sites | 0.044 | Intercept | -2.129 | [-2.377, -1.881] | <0.001 |
|  |  |  | Slope | 0.37 | [0.031, 0.043] | <0.001 |
|  | IBZ only | 0.012 | Intercept | -1.321 | [-1.687, -0.956] | <0.001 |
|  |  |  | Slope | 0.022 | [0.009, 0.035] | 0.001 |
| Normal sinus beats | All sites | 0.055 | Intercept | -2.576 | [-2.805, -2.347] | <0.001 |
|  |  |  | Slope | 0.069 | [0.059, 0.079] | <0.001 |
|  | IBZ only | 0.011 | Intercept | -1.781 | [-2.137, -1.425] | <0.001 |
|  |  |  | Slope | 0.037 | [0.015, 0.060] | 0.001 |
| Group I VPBs | All sites | 0.014 | Intercept | 0.481 | [-0.825, 1.787] | 0.469 |
|  |  |  | Slope | 0.042 | [-0.005, 0.090] | 0.082 |
|  | IBZ Only | 0.018 | Intercept | 0.897 | [-0.345, 2.139] | 0.154 |
|  |  |  | Slope | -0.059 | [-0.165, 0.046] | 0.266 |

# Supplementary Figures

**
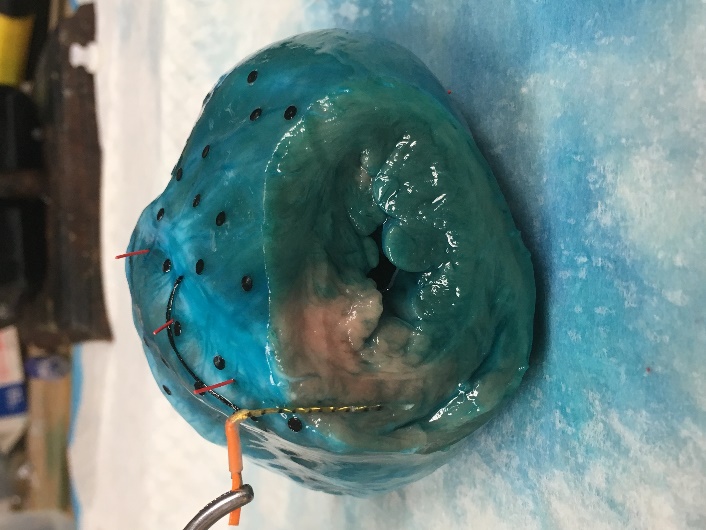
**

Supplementary Figure 1. Ischemic zone identified by injecting fast green dye at the end of experiments.


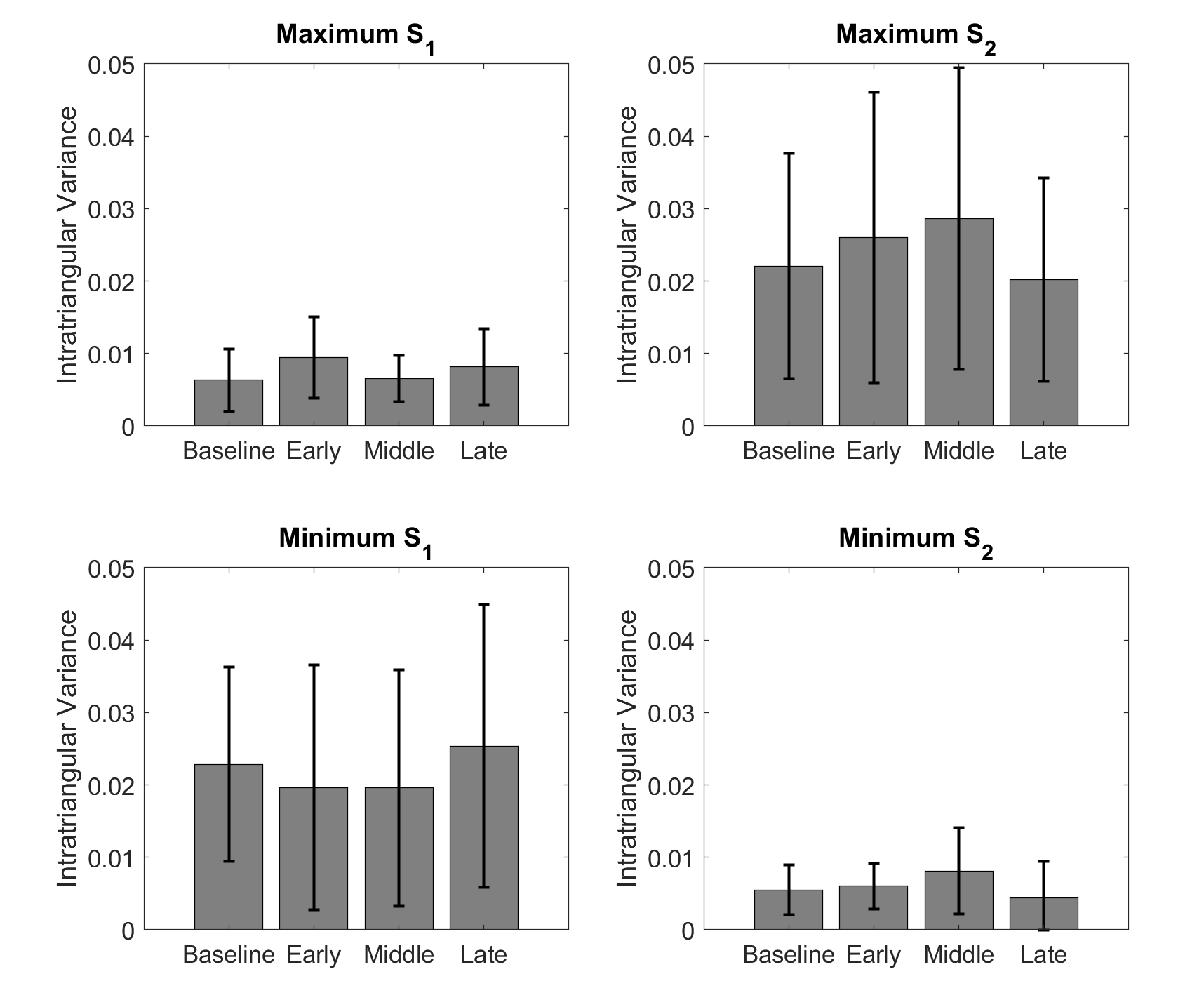


Supplementary Figure 2. Deformation homogeneity among small component triangles of large (~8mm) triangles in the IBZ. *S_1_* and *S_2_* are principal strains (*S_1_* < *S_2_*). Intra-triangular variance does not differ significantly between baseline and three ischemic conditions (all p>0.05, repeated measured ANOVA).


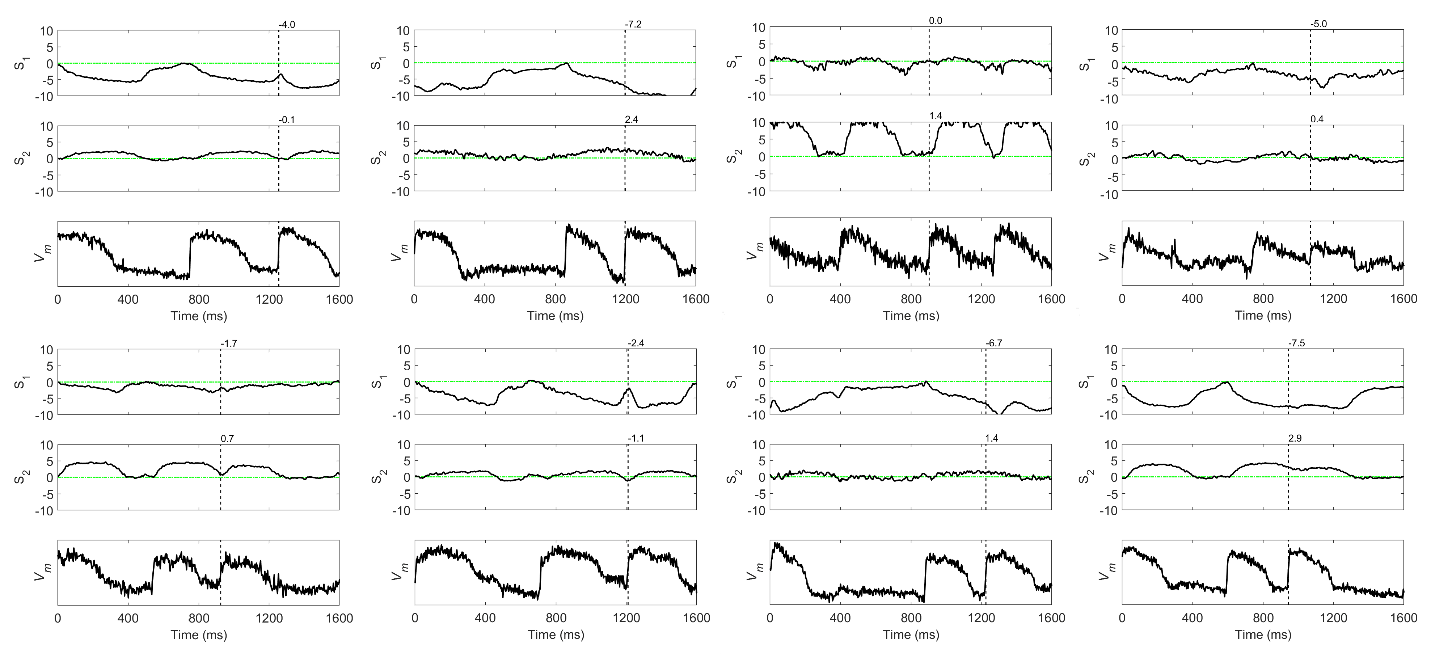


Supplementary Figure 3. Additional examples of principal strains (in %) and *V_m_* at ectopic focal sites. Vertical dashed lines indicate the moment of ectopic depolarization (maximum d*V_m_*/d*t*). Values on top of vertical dashed lines are the strains at depolarization time.


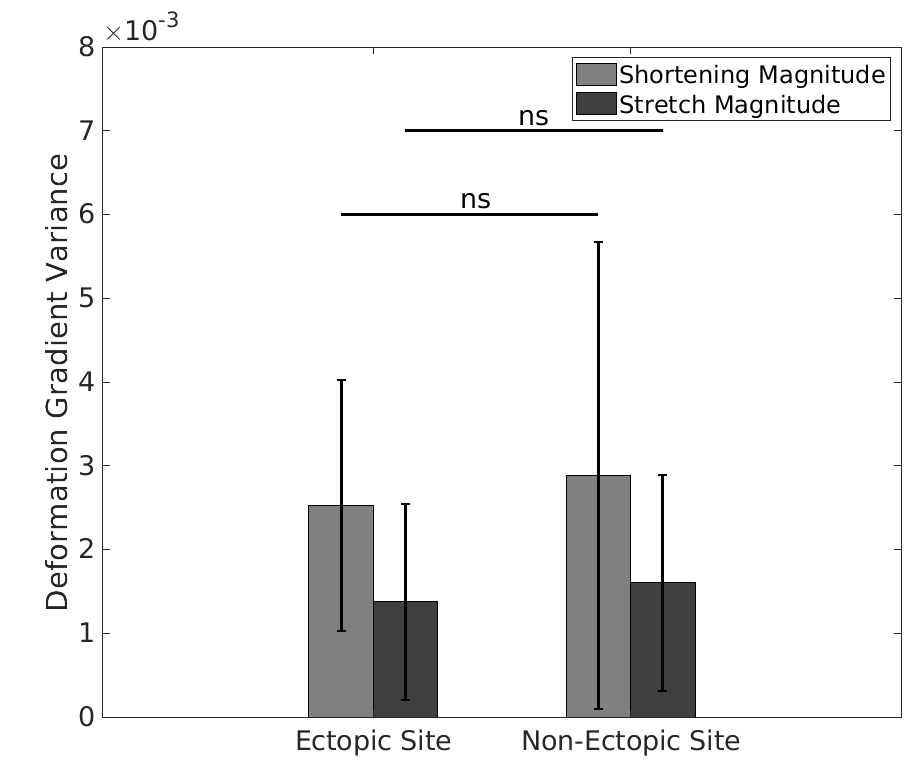
Supplementary Figure 4, deformation magnitude heterogeneity at ectopic sites vs. matched non-ectopic sites. The gradients of deformation magnitudes were computed between ectopic/non-ectopic sites and their 2 or 3 adjacent triangles. The variance of deformation gradients does not differ significantly between ectopic site and non-ectopic site (p=0.295 and p=0.124 for shortening and stretch magnitudes, respectively. Paired t-test).

# Supplementary Methods

## Heart Preparation

Isolated hearts from 16 pigs of either sex, weighting 25-35 kg were assigned to two groups of eight: left ventricular working (LVW) and non-working (NW). Three additional Langendorff-perfused hearts were used as a sham group and three additional working hearts were used to evaluate homogeneity of deformation. Modified Krebs-Henseleit solution (composition given by Chinchoy et al.^1^) was used as perfusate in all groups. Anesthesia, heart excision and aortic cannulation were similar to our previous publication.^2^ Briefly, anesthesia was induced with intramuscular telazol (4.4mg/kg), xylazine (2.2 mg/kg) and atropine (0.04 mg/kg) and maintained with isoflurane in 100% oxygen. 500 IU/kg heparin was given intravenously. The chest was opened, the aorta was clamped, and the heart was rapidly excised and placed in a bath of ice cold saline. Euthanasia was by exsanguination secondary to heart excision. The aortic root was cannulated and the coronary arteries were perfused with chilled perfusate. In NW and LVW groups, to measure mechanical deformation and correct motion artifact, 20-30 black circular markers (2 mm diameter, ~8 mm spacing) were glued to the anterior ventricular epicardium with tissue glue (1469SB, 3M Vetbond). Most markers were on the LV, but in 10 animals, 3-5 markers were also placed on the RV adjacent to its anterior insertion.

Hearts were then mounted in the perfusion apparatus and a polypropylene foam support was placed against the posterior side to reduce rigid-body swinging.

NW and sham hearts were perfused in Langendorff mode at 200mL/min with warm (37±1 °C) perfusate saturated with carboxygen (95%/5% O_2_/CO_2_). Once warm, the hearts were defibrillated and (-)-blebbistatin (MedChem Express) was added to the perfusate (10 µmol/L final concentration) to suppress contraction. Thirty minutes were allowed for stabilization and blebbistatin incubation. The hearts were then stained with the potentiometric dye di-4-ANEPPS (di4) (Biotium) for imaging membrane potential (*V_m_*) propagation. To stain, 75 mL of oxygenated di4-containing perfusate was recirculated through the coronaries at 100 mL/min. Two 0.25 mL boluses of di4 stock solution (1 mg/mL in dimethyl sulfoxide) were added to the perfusate with final di4 concentrations of 7 and 14 µmol/L. The first bolus was added, and once fluorescence intensity plateaued, the second bolus was added. The staining procedure was completed within 10 minutes. Blebbistatin loading, di4 staining and all subsequent procedures were performed in the dark or under red light to prevent photobleaching.

The LVW preparations were similar to our previous publication^3^ except that the left atrium was cannulated through the pulmonary veins instead of the appendage. Briefly, the heart was excised with the lungs and immediately immersed in 3 L chilled saline solution. The aortic root was cannulated and the coronary arteries were flushed with chilled Krebs-Henseleit solution. The lungs were cut away and the left atrium was cannulated through the opening of the pulmonary veins. The aortic and atrial cannulas were connected to afterload and preload columns, respectively. The hearts were initially perfused in Langendorff mode by closing valves to the afterload and preload chambers and retrogradely pumping perfusate to the aortic cannula at 200 ml/min. Once warm, hearts were defibrillated and the perfusion system was switched to LVW mode by opening the afterload and preload valves. The preload and afterload pressures were set to 5-20 mmHg and 45-55 mmHg, respectively. The hydrostatic pressure in the afterload chamber drives the coronary flow continuously, independently of LV pumping function. The perfusate in the preload and afterload chambers was maintained at 37±1 °C and saturated with carboxygen. Contraction was *not* suppressed with blebbistatin. The hearts were allowed to stabilize for 30 minutes (the same time allowed for blebbistatin incubation in the NW/sham hearts). Perfusion was then temporarily switched back to Langendorff mode and di4 was loaded as described above.

## Electromechanical Optical Mapping

In NW and LVW hearts, anterior LV epicardial electrical activity and mechanical deformation were measured with our recently developed electromechanical optical mapping system.^3^ This system uses a combination of motion tracking and excitation ratiometry to obtain *V_m_* signals in beating hearts. Briefly, the motion of the epicardial markers was tracked. Markers close to the edge of the heart’s silhouette could leave the field of view during the acquisition or move primarily in a direction normal to the mapping camera plane. Such markers were deemed untrackable. Tissue motion was characterized by interpolating marker displacement. Excitation ratiometry was used for further motion artifact correction. Di4 was excited by 450 nm or 505 nm light-emitting diodes (LED) (Luxeon Z royal blue and cyan, respectively, Lumileds). The emitted fluorescence was filtered (645±37.5 nm) and recorded with a mapping camera (iXon DV-860DC-BV, Andor). Excitation light wavelength was switched with each camera frame. The blue-elicited fluorescence signal was insensitive to *V_m_* and thus contained only motion artifacts. Cyan-elicited fluorescence was sensitive to *V_m_* and thus contained both motion artifact and *V_m_*. By taking the ratio of blue- to cyan-elicited signals, the common motion artifact was suppressed and *V_m_* was recovered. The camera acquired at 750 frames/sec for an effective frame rate of 375 frames/sec after ratiometry. Three additional geometry cameras (Genie HM640, Teledyne DALSA) were mounted on the sides and top of the mapping camera and synchronized with the blue-illuminated frames. These cameras tracked the epicardial markers in three-dimensional space, enabling mapping of epicardial mechanical deformation.

Due to a software limitation, the optical mapping system could record continuously for a maximum of 20 minutes. Consequently, longer acquisitions were interrupted by short pauses (<60 seconds) for saving data and restarting. The electrical mapping system was able to record continuously.

## Identification of Ectopic Events

Ectopic events with a ventricular source were identified using electrograms collected from the subepicardial electrodes in the heart’s four chambers. Activation time was defined as the maximum negative peak in an electrogram’s first derivative.^4^ The delay (*T_A-V delay_*) between activation in the RA signal (gray dashed lines in Figure 2) and activation in the LV signal of a normal sinus beat was identified from baseline data. A time window (gray shading in Figure 2) from *(1-5%)T_A-V delay_* to *(1+5%)T_A-V delay_* was created after each activation in the RA signal. Any LV activation outside of this window was identified as a potential ventricular ectopic event (e.g. the 2^nd^ activation in Figure 2C). If the activation rate changed during the recording, *T_A-V delay_* was updated.

## Ectopic Activations with Undetermined Origin

Ectopic activations were deemed to have an undetermined origin if (1) the earliest activation was on the edge of the expanded optical mapping region, suggesting that it propagated from outside, (2) it occurred during the pauses between optical mapping acquisitions, (3) the earliest activation was in the posterior needle electrode, or (4) the earliest activation was on the edge of the marked region and could not be detected in the expanded mapping region because of strong residual motion artifact.

## Deformation Homogeneity along the Ischemic Border Zone

In our previous publication,^3^ using nonischemic hearts, we identified 8 mm marker spacing as optimal to achieve homogeneous strain within each triangle without blocking optical access to the epicardium with excessive markers. In the present study, we used three pig hearts to validate this spacing within the ischemic border zone where additional heterogeneity might be expected. The hearts were prepared the same as the LVW hearts, except we used a much smaller marker spacing (3-4 mm). The triangles lying within the IBZ were identified by injecting fast green dye after the experiment. We grouped sets of 3 IBZ triangles into large triangles with ~8mm marker spacing. We analyzed marker motion data for one cardiac cycle recorded before occlusion (baseline) and at 3 times after LAD occlusion (20±5 min (early), 30±5 min (middle), and 40±5 min (late) post-occlusion). We found the maximum and minimum values of *S_1_* and *S_2_* for each small triangle over each cardiac cycle. We then computed the variance of these strains among the 3 subtriangles in each large triangle. There were a total of 17 large triangles (5-7 per heart), which we treated as independent because each was in a different position relative to its border zone. By repeated measures ANOVA, there were no significant differences in intra-triangular strain variance among the 4 groups (baseline, early, middle, late; Supplementary Figure 2). Thus, the assumption of homogeneous strain for triangles with ~8 mm marker spacing remains valid in the ischemic border zone during early acute ischemia.

# Supplementary References

1. Chinchoy E, Soule CL, Houlton AJ, Gallagher WJ, Hjelle MA, Laske TG, Morissette J, Iaizzo PA. Isolated four-chamber working swine heart model. *Ann Thorac Surg*. 2000;70:1607–1614.

2. Qin H, Kay MW, Chattipakorn N, Redden DT, Ideker RE, Rogers JM. Effects of heart isolation, voltage-sensitive dye, and electromechanical uncoupling agents on ventricular fibrillation. *Am J Physiol Heart Circ Physiol*. 2003;284:H1818-1826.

3. Zhang H, Iijima K, Huang J, Walcott GP, Rogers JM. Optical Mapping of Membrane Potential and Epicardial Deformation in Beating Hearts. *Biophys J*. 2016;111:438–451.

4. Cantwell CD, Roney CH, Ng FS, Siggers JH, Sherwin SJ, Peters NS. Techniques for automated local activation time annotation and conduction velocity estimation in cardiac mapping. *Comput Biol Med*. 2015;65:229–242.
